# Supplementary figures and images for: Cyclin-Dependent Kinase Subunit 2 (CKS2) as a Prognostic Marker for Stages I–III Invasive Non-Mucinous Lung Adenocarcinoma and Its Role in Affecting Drug Sensitivity
Source: Cells. 2022 Aug 22;11(16):2611. doi: 10.3390/cells11162611 (PMC9406629; doi:10.3390/cells11162611)

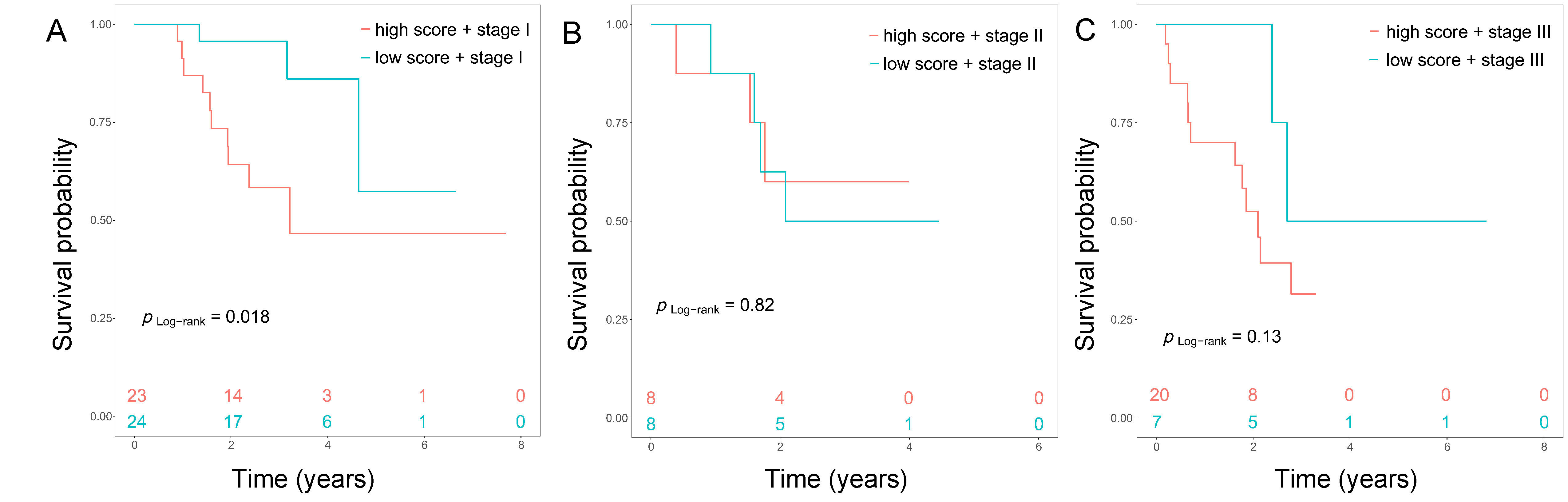

Supplement: Supplementary file 1 [file cells-11-02611-s001.zip › cells-1845645-supplementary.jpg]
